# Characterisation of STEC and other diarrheic *E. coli* isolated on CHROMagar™STEC at a tertiary referral hospital, Cape Town

**DOI:** 10.1186/s12866-018-1195-7

**Published:** 2018-06-08

**Authors:** John Bosco Kalule, Karen H. Keddy, Mark P. Nicol

**Affiliations:** 1 Division of Medical Microbiology, Department of Pathology, Faculty of Health Sciences, University of Cape Town and National Health Laboratory Services, Cape Town, South Africa; 2 Centre for Enteric Diseases, National Institute for Communicable Diseases, Johannesburg, South Africa; 3 Faculty of Health Sciences, University of the Witwatersrand, Johannesburg, South Africa

**Keywords:** CHROMagar™STEC, Tellurite resistant diarrhoeic *E. coli*, STEC, Shiga toxin, Africa

## Abstract

**Background:**

Shiga toxin producing *E. coli* (STEC) is an emerging zoonotic pathogen that can cause acute renal failure, especially in children. Clinical microbiology laboratories may fail to detect STEC and other diarrhoeic *E. coli* unless purposive rigorous screening procedures are followed using appropriate diagnostic technology; CHROMagar™STEC has rarely been used for isolation of African diarrhoeic *E. coli* hence characteristics of isolates on this medium are not yet fully understood. This study aimed to determine the prevalence and characteristics of STEC and other diarrhoeic *E. coli* isolated on CHROMagar™STEC from stool samples submitted to the microbiology laboratory of a South African public sector tertiary care hospital.

**Results:**

In total, 733 stool samples were tested. Of these, 4.5% (33/733) possessed diarrhoeic *E. coli.* Of the diarrheic *E. coli,* 5/33 (15.2%) were STEC, 15/33 (45.5%) EAggEC, 6/33 (18.2%) atypical EPEC, 5/33 (15.2%) typical EPEC, and 1/33 (3%) DAEC. None of the STEC isolates had been identified by routine testing (based on using sorbitol media to test for *E. coli* O157: H7 strains and not the other STEC) in the laboratory. Of the 33 strains, 55% (95% CI = 40.8–72.7) showed resistance to ampicillin.

**Conclusions:**

CHROMagar™STEC enabled detection of tellurite - resistant diarrhoeic *E. coli* that would be missed using routine methods. Further studies are needed to determine the proportion and characteristics of those which might have been missed using this approach.

## Background

Shiga toxin-producing *E. coli* (STEC) are important causes of diarrhoea, haemorrhagic colitis, bloody diarrhoea, and haemolytic-uraemic syndrome (HUS) [1]. The use of sorbitol MacConkey to screen for STEC has limitations due to its inability to detect emerging sorbitol-fermenting non-O157 STEC and sorbitol-fermenting O157 strains [2]. Indeed, new chromogenic media, such as CHROMagar™STEC, have been developed to identify both O157 and non-O157 STEC with some targeting only specific STEC serotypes [3].

CHROMagar™STEC is a screening medium with a STEC recovery rate of 70%, which selects for tellurite-resistant STEC [3]. The STEC serotypes commonly associated with severe disease globally (O26, O45, O103, O111, O121, O145, and O157) [4] can readily be isolated on CHROMagar™STEC because they are commonly tellurite resistant [5]. Tellurite resistance is defined as growth at a minimum inhibitory concentration of 2.5 μg/ml to ≥20 μg/ml), [6] however the concentration of potassium tellurite in CHROMagar™STEC is proprietary. On this agar, the STEC serotypes that are commonly associated with severe human infections (O26, O45, O103, O111, O121, O145, and O157) [4, 7] form mauve colonies, other Enterobacteriaceae form blue or colourless colonies, while the growth of gram-positive bacteria is inhibited (http://www.chromagar.com/products-chromagar-stec-focus-on-stec-e-coli-51.html#.WNbR4vnyu01). On this media, tellurite-resistant *E. coli* pathotypes including STEC, form mauve coloured colonies [8]. The chemistry behind the formation of the mauve coloured colonies is proprietary information. Given that the routinely used culture method for STEC is based on sorbitol fermentation, and intended for *E. coli* O157: H7, it would miss sorbitol fermenting STEC.

Even though diarrhoea is not commonly treated using antibiotics, antibiograms of diarrhoeic *E. coli* can be utilised for sentinel surveillance of antimicrobial resistance [9]. Even though diarrhoeic *E. coli* infections are usually self-limiting, persistent and invasive infections, especially in the immunocompromised persons, may necessitate the use of antibiotics [10].

In an earlier study which evaluated STEC diagnostic technology on African isolates, an in-house-developed duplex real-time PCR assay for detection of *stx*_*1*_ and *stx*_*2*_ was validated and tested on diarrhoeic stool samples and then used as a reference standard to assess the performance of CHROMagar™STEC [11]. A related study used quantitative proteomics to show that mauve colonies on CHROMagar™STEC produced tellurite resistance proteins TerA, TerE, TerC, TerB, TerD, TerW, and TerZ [12]. This study purposed to determine the characteristics (pathotypes, serotypes, antibiogram, and sorbitol fermentation ability) of African isolates that grew on CHROMagar™STEC with and without TSB enrichment of stool samples submitted to the microbiology laboratory of a South African public sector tertiary hospital.

## Methods

### Sample collection and processing

**Fig. 1:**
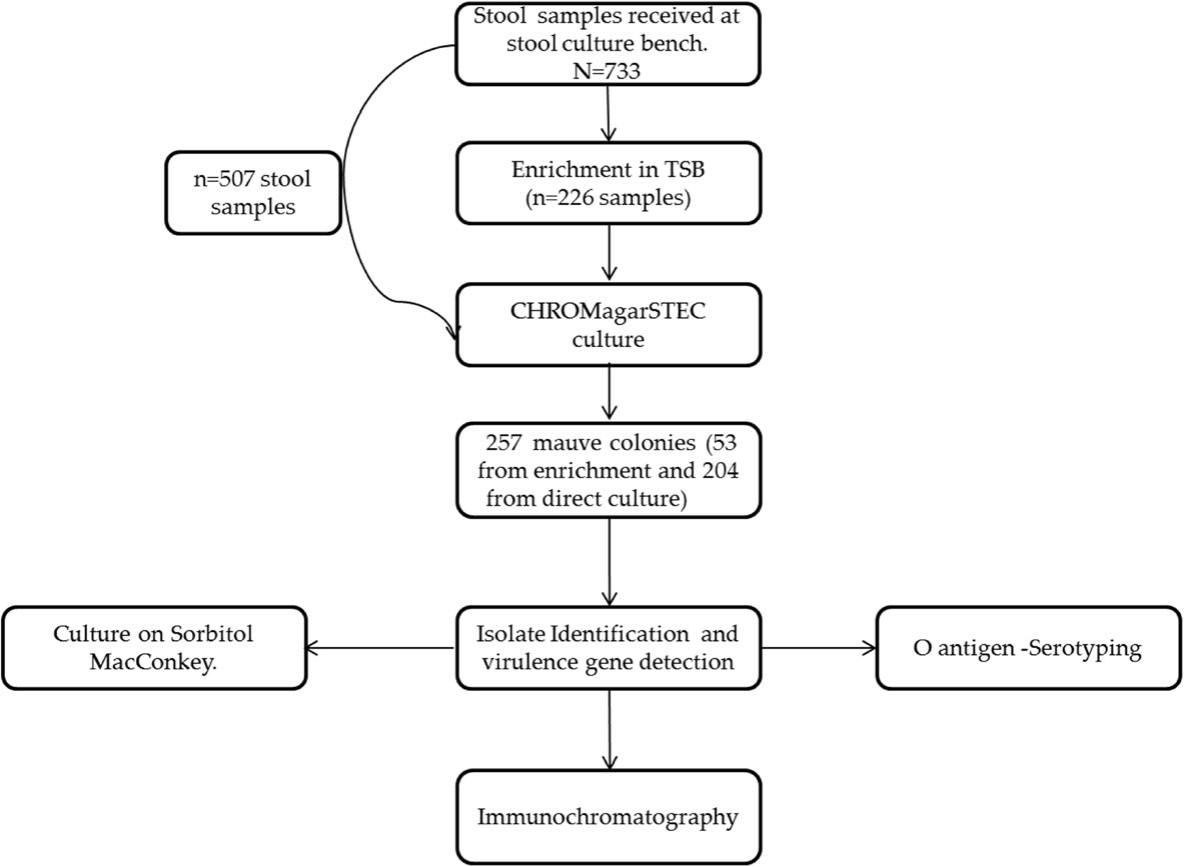
Summary of the methods used in this study

This study is part of a larger study on STEC in human and non-human sources in Cape Town. At the National Health Laboratory Services (NHLS) laboratories at Groote Schuur Hospital (GSH), stool specimens are not routinely screened for diarrhoeic *E. coli*. Physicians are advised to contact the laboratory within seven days if there is a clinical suspicion of diarrhoeic *E. coli* infections such as STEC. Diarrhoea, in this study, was defined as more than three loose stools in less than 24 h. Freshly collected diarrhoeic stool specimens (in a screw-capped stool collection container and delivered in a temperature regulated box) submitted to the NHLS at GSH, Cape Town, between September 2014 and May 2015 for microbiological testing (as part of standard patient care) were screened for diarrhoeic *E. coli*. All the stool samples from diarrhoea samples were tested irrespective of the age of the patient as recommended by the Centre for Disease Control (CDC) [13]. In order to isolate tellurite resistant STEC and other diarrheic *E. coli*, samples were either inoculated directly onto CHROMagar™STEC (CHROMagar Microbiology, Paris, France) or subjected first to overnight enrichment in Tryptic Soy Broth (TSB; Oxoid, Basingstoke, U.K) before inoculation onto CHROMagar™STEC (Fig. 1). Mauve colonies, indicative of growth and therefore tellurite resistance, were visually identified and selected (a maximum of 5 per sample) for characterisation. Pathotypes and serotypes were determined as previously described [11].

Briefly, to dertermine the pathotypes, end point PCR reactions targeting the fimbrial adhesion gene (*daaC*) for diffusely adherent *E. coli* (DAEC), the anti-aggregation protein transporter gene (*aat*) for enteroaggregative *E. coli* (EAggEC), heat-stable (*ST*) and heat-labile (*LT*) enterotoxin genes of enterotoxigenic *E. coli* (ETEC), the invasive plasmid antigen gene (*ipa*) for enteroinvasive *E. coli* (EIEC), and the intimin coding gene (*eae*) for enteropathogenic *E. coli* (EPEC) were done as previously described [14]. An in-house developed duplex real-time PCR assay was used to screen for stx_1_ and stx_2_. The *stx* + *E. coli* isolates were tested for Shiga toxin production using the immunocard STAT® EHEC (Meridian Biosciences Inc., Cincinnati, Ohio, United States). Subsequently, clinical data were reviewed for all diarrhea cases that carried STEC to assess for presentation of typical haemolytic uremic syndrome (tHUS).

To determine the serotypes*,* serology was done to detect the somatic O- antigens using reagents supplied by the Statens Serum Institut, Copenhagen, Denmark and the tube agglutination method as previously described [15]. Serology to detect the H-antigens was not done.

### Culture characteristics on sorbitol MacConkey

Briefly, mauve isolates identified as *E. coli* using the VITEK 2 system (bioMérieux, USA) were cultured on Sorbitol MacConkey and incubated for 18 h at 35 °C. Colourless colonies were considered to be non-sorbitol fermenting. Antimicrobial susceptibility testing.

Isolates were tested for susceptibility to 19 antibiotics following the Clinical Laboratory Standards Institute guidelines (CLSI, 2015). Since VITEK offers a limited MIC range, we further re-tested these isolates using the broth microdilution based GNX2F sensititre MIC plate (Thermo-scientific, USA) to confirm the MICs observed using VITEK 2 systems. MIC values from broth microdilution were used as the final reference for classification as resistant (R), intermediate (I) or susceptible (S). *E. coli* ATCC 25922 was used as the control strain. The antibiotics included in the broth microdilution panel on the GNX2F sensititre plate were: amikacin (AMK), aztreonam (AZT), cefepime (FEP), cefotaxime (CTX), ceftazidime (CAZ), ciprofloxacin (CIP), colistin (COL), doripenem (DOR), doxycycline (DOX), ertapenem (ETP), gentamicin (CIN), imipenem (IMP), levofloxacin (LEV), meropenem (MEM), minocycline (MIN), piperacillin-tazobactam (TZP), polymixin B (POL), ticarcillin-clavulanic acid (TIM), tigecycline (TGC), tobramycin (TOB), and trimethoprim – sulfamethoxazole (SXT).

MIC data were entered into WHONET 5.6 software. Resistance to third-generation cephalosporins and or carbapenems was confirmed using the ESB1F sensititre MIC plates (Thermo-scientific, USA).

### Data analysis

Data on cultural characteristics, serotypes, and pathotypes was entered into Microsoft Office Excel 2016 (Microsoft Corporation, USA) software, coded and exported to STATA version 12 for analysis. Further analysis was done using the R-statistical package [16]. Results were entered in WHONET 5.6 (World Health Organization, Geneva) and interpreted in accordance with current Clinical Laboratory Standards Guidelines [17].

## Results

The mean age of the patients whose stool samples possessed diarrheic *E. coli* was 20.6 (± 17.4) years (Table 1). Of the 733 specimens, 507 were directly inoculated on CHROMagar™STEC while 226 were first enriched in TSB before inoculation on CHROMagar™STEC. Of 733 specimens screened on CHROMagar™STEC, 257 (35%) yielded mauve colonies. Mauve colonies were obtained from 40% (204/507) of the directly inoculated specimens, and 24% (53/226) of stool samples that were enriched first in tryptic soy broth (*p* = 0.001). Of the mauve colonies obtained from direct culture, 5.9% (12/204)were diarrhoeic *E. coli* while 39.6% (21/53) of the mauve colonies obtained following enrichment in TSB were diarrhoeic *E. coli*.

**Table 1:** Growth and Virulence properties of diarrhoeic *E. coli* that formed Mauve colonies on CHROMagar™STEC

Isolate No.	HUS	Bloody diarrhoea	Serotype	Virulence genes.	Pathotype	Immunoassay	TSB enrichment	Sorbitol
271		–	O55	*eae,bfp*	Typical EPEC		–	–
724		–	O55	*eae,bfp*	Typical EPEC		+	–
722.1		–	O55	*eae,bfp*	Typical EPEC		+	–
279		–	O55	*eae, bfp*	Typical EPEC		–	–
344		–	O55	*eae, bfp*	Typical EPEC		+	–
345.1		–	O55	*eae, bfp*	Typical EPEC		+	+
291		–	O182	*eae*	Atypical EPEC		–	+
286		–	O111	*eae*	Atypical EPEC		+	+
600		+	Not Identified	*eae*	Atypical EPEC		+	+
19		+	O26	*eae*	Atypical EPEC		–	–
284		–	O25	*eae*	Atypical EPEC		–	+
15.2		+	O26	*eae*	Atypical EPEC		–	–
63.2		–	O1	*daaC*	DAEC		–	+
689		–	O175	*aat*	EAggEC		+	–
326		–	O16	*aat*	EAggEC		+	–
231		–	O104	*aat*	EAggEC		–	–
229.1		–	O104	*aat*	EAggEC		–	–
473		–	O33	*aat*	EAggEC		+	+
371.1		+	O25	*aat*	EAggEC		+	–
336		–	O175	*aat*	EAggEC		+	+
229		–	O104	*aat*	EAggEC		–	–
733		–	O104	*aat*	EAggEC		+	–
502		–	O9	*aat*	EAggEC		+	+
688		–	O3	*aat*	EAggEC		+	+
480		–	O8	*aat*	EAggEC		+	+
250		–	O175	*aat*	EAggEC		–	+
207		–	O104	*aat*	EAggEC		–	+
696		–	O175	*aat*	EAggEC		+	+
598	–	–	O186	*stx*_*1*_ *, eae*	STEC	–	+	+
29.5	–	–	O186	*eae, stx*_*1*_	STEC	–	+	+
602	–	–	O101	*eae, stx*_*1*_ *, stx*_*2*_	STEC	–	+	–
232.1	–	–	Not identified	*stx*_*1*_	STEC	Shiga toxin 1	+	+
73		–	Not identified	*stx*_*1*_	STEC	Shiga toxin 1	+	

EAggEC-enteroaggregative *E. coli, STEC-*Shiga-toxin producing *E. coli, EPEC-*enteropathogenic *E. coli, DAEC-*diffusely adherent *E. coli.* Fimbrial adhesion gene *(daaC),* the anti-aggregation protein transporter gene *(aat),* heat-stable *(ST)* and heat-labile *(LT)* enterotoxin genes*,* the intimin coding gene *(eae)* and the bundle-forming pili gene *(bfp),* Shiga toxin 1 *(stx*_*1*_*)* and 2 *(stx*_*2*_*)*

Tellurite resistant diarrhoeic *E. coli* were therefore obtained from 5% (33/733) of the stool specimens.

The growth and virulence attributes of the isolates that formed mauve colonies on CHROMagar™STEC are shown in Table 1**.** Of the 33 diarrhoeic *E. coli*, 64% (21/33) were obtained following enrichment in TSB while 36% (12/33) were obtained without enrichment (*p* = 0.004). Of these, 15.2% (5/33) were STEC, 45.5% (15/33) were EAggEC, 18.2% (6/33) were atypical EPEC, 15.2% (5/33) were typical EPEC, and 3% (1/33) were DAEC. No EIEC or ETEC were detected.

Among the diarrhoea cases that carried STEC, co-infection with other bacterial pathogens (as evidenced from the routine microbiology testing results) was not detected; and none presented with HUS.

A total of 16 different serotypes were identified, of which **s**erotypes O104 (5/33, 15.2%) and O55 (6/33, 18.2%) were most common. All the *E. coli* isolates that belonged to serotypes O104 and O55 were EAggEC and EPEC respectively.

**Fig. 2:**
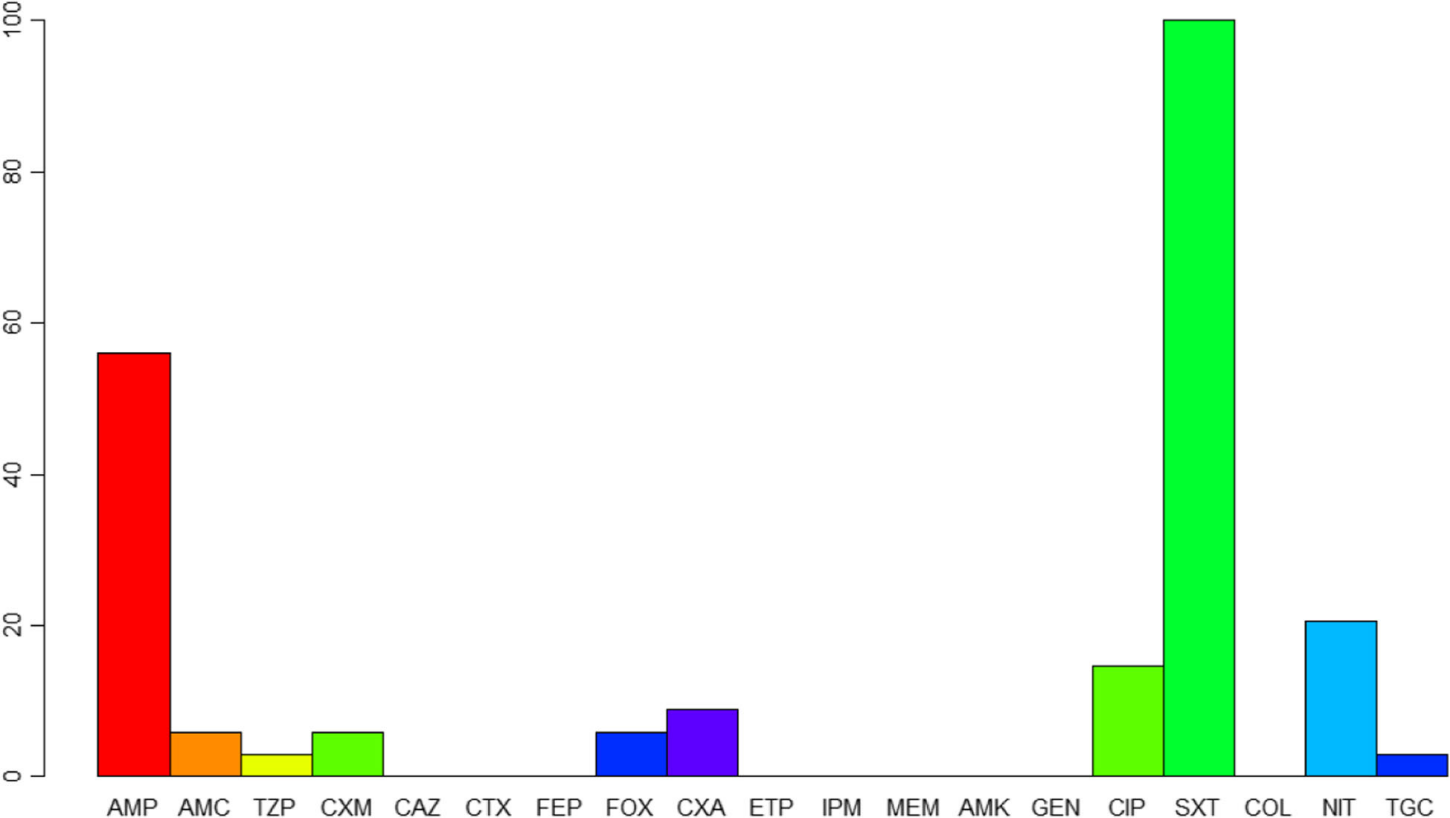
Antimicrobial susceptibility patterns of diarrheic *E. coli* isolated on CHROMagar™STEC; AMP-ampicillin, AMC-amoxicillin clavulanate, CXM-cefuroxime, CAZ-ceftazidime, CTX-cefotaxime, FEP-cefepime, FOX-cefoxitin, CXA-cefuroxime axetil, ETP- ertapenem, IPM-imipenem, MEM-meropenem, AMK-amikacin, GEN-gentamicin, CIP-ciprofloxacin, SXT- trimethoprim-sulfamethoxazole, COL-colistin, NIT-nitrofurantoin, TGC-tigecycline

Of the thirty-three diarrhoeic *E. coli*, 54.5% (18/33) were resistant to AMP, 3% (1/33) to TZP, 6.1% (2/33) to CXM, 3% (1/33) to FOX, 21.2% (7/33) to NIT, 3% (1/33) to TGC and 3% (1/33) to ciprofloxacin (Fig. 2). All the isolates were resistant to SXT.

Only three isolates were multidrug-resistant, one of which (EAggEC, serotype O25) was resistant to six antibiotics while the atypical (serotype O182) and typical EPEC (serotype O55) were resistant to three and four antibiotics respectively. The commonest resistance profile was resistance to only ampicillin (Table 2). All the STEC were susceptible to all the antibiotics except SXT.

**Table 2:** Resistance profiles of Diarrhoeic *E. coli* obtained with and without enrichment

Number of isolates	Resistance Profile
18	AMP
1	AMP AMC CXA
1	AMP CXM CXA FOX
1	AMP AMC TZP CXM CXA FOX

*AMP* ampicillin, *AMC* amoxicillin clavulanate, *CXA* cefuroxime axetil, *CXM* cefuroxime, *FOX* cefoxitin, *TZP* tazobactam piperacillin, *EAggEC* enteroaggregative *E. coli*, *EPEC* enteropathogenic *E. coli*

Of the 33 tellurite-resistant diarrhoeic *E. coli*, 45.5% (15/33) were non – sorbitol fermenting. Of the 15, seven were EPEC (46.7%) while seven (46.7%) were EAggEC. Of the five STEC isolates, only one was non-sorbitol fermenting.

## Discussion

The main findings of this study were; (1) Tellurite resistant diarrhoeic *E. coli* were obtained from 5% (33/733) of the stool specimens and included; five STEC, twelve EPEC, one DAEC, and fifteen EAggEC strains, (2) All the diarrhoeic *E. coli* isolated in this study were resistant to trimethoprim-sulphamethoxazole while 55% (18/33) were resistant to ampicillin.

### Detection of diarrhoeic *E. coli* in stool

The inhibitory action of potassium tellurite on coliforms was first reported by Fleming in 1940 [18]. Tellurite-containing media have been routinely used to screen for STEC [19], but not the other *E. coli* pathotypes. However, Hirvonen et al., 2012 reported the formation of mauve colonies by other tellurite resistant diarrhoeic *E. coli* on this medium [8]. Of the 733 stool samples processed in this study, 33 (5%) contained tellurite-resistant diarrhoeic *E. coli*. However, tellurite-containing media have limited ability to select for STEC. In a study conducted on STEC from human, animals, and food in Austria, the prevalence of tellurite resistance amongst STEC was 26% - therefore 74% of the STEC would not be detected by the tellurite-containing medium. [20]

Tellurite resistance and antimicrobial resistance are often observed in highly pathogenic diarrhoeic *E. coli* strains [20]. Commercial selective media such as CHROMagar™STEC and Sorbitol MacConkey with cefixime and potassium - tellurite use this property to select STEC O157: H7 and other pathogenic STEC. There was a relatively low prevalence (5%, 33/733) of tellurite-resistant diarrhoeic *E. coli* in stool samples submitted to NHLS, GSH. We were unable to determine the prevalence of tellurite susceptible STEC in this study; this is because these would not grow on CHROMagar™STEC.

### STEC

Five STEC were detected in this study. All the five were detected after enrichment in TSB, and none was obtained by directly streaking on CHROMagar™STEC. Since the other diarrheic *E. coli* pathotypes were detected with and without enrichment, it can, therefore, be speculated that TSB enrichment is more beneficial for STEC isolation relative to the other diarrheic *E. coli* pathotypes. Overall, the number of diarrheic *E. coli* obtained after enrichment in TSB was significantly higher than the number obtained without (*p* = 0.004). This may suggest that enrichment in TSB may be a beneficial step as regards recovering STEC (and other pathotypes) from a stool sample. The STEC diagnostic strategy routinely employed by the NHLS clinical laboratory at the GSH targets only O157 STEC and is based on the non-sorbitol fermenting attribute of STEC O157: H7. Therefore, this strategy would miss the non-O157 STEC and sorbitol-fermenting *E. coli* O157. Of the five STEC reported in this study, only one was non-sorbitol fermenting and belonged to serotype O101. The five non- O157 STEC that we report in this study would have been missed (four because they were sorbitol fermenting and one because it was not O157) given that the routinely used method is based on absence of sorbitol fermentation and targets only *E. coli* O157:H7. Only two of the five STEC tested positive for Shiga toxin production by immunoassay because the other three did not express the *stx* genes.

The relatively low rate of STEC isolation in this study could be due to several reasons: (1) The *stx* primers may not have been suitable for detection of all the *stx* gene variants [21]. (2) The tellurite susceptible STEC could not be isolated on CHROMagar™STEC. (3) This study had a short sampling frame and not all children presenting with diarrhoea may have had a stool specimen taken. (4) There could have been a low prevalence of STEC in stool samples processed, especially given that the stool specimens were not from patients presenting with bloody diarrhoea, or HUS. There are higher chances of recovering STEC from the stool of patients with bloody diarrhoea or HUS as opposed to those without [22].

In South Africa, as in other African countries, there may be a lower prevalence of STEC as compared to America and Europe. This may be related to the type of diet given to ruminants in America and Europe that favour the proliferation of STEC in cattle-the primary ruminant reservoirs for STEC [23].

It is important to detect STEC in stool because the use of antibiotics such as the quinolones leads to bacterial lysis and toxin release which increases the chances of HUS in infected patients [23]. Four of the five STEC detected in this study carried the *stx*_*1*_ genes. Possession of *stx*_*2*_ genes has been associated with the more severe form of illness [24]. Also, we detected *eae* in three of the five STEC, while two did not possess the *eae* gene. The *eae* gene which is located in the locus of enterocyte effacement (LEE) codes for the intimin protein which is necessary for the formation of attaching-effacing lesions in the intestinal tract [25]. STEC that carry *eae* (LEE-positive STEC) have been shown to cause more severe disease.

Per the manufacturer of CHROMagar™STEC, the commonly encountered STEC serotypes should form mauve coloured colonies on this medium. However, the serotypes were categorised as “common” (O157, O26, O45, O145, O111, O121, and O103) based on outbreaks that occurred in developed countries. In this study, STEC belonged to serotypes O101 and O186. These serotypes were not detected in an earlier study conducted at the NICD (2006 to 2009) which screened 2378 diarrhoeic *E. coli* isolates. In that study, STEC in stool had not been purposively targeted (and thus CHROMagar™STEC was not used) but was an incidental finding among EPEC (the strains carried the eae gene) that had been sent to the central public health laboratory for serotyping. The 14 STEC identified in that study belonged to serotypes O4, O5, O21, O26, O84, O111, and O157 [26]. Other related studies reported STEC serotypes O8 and O9 in pigs in South Africa [27]. We did not detect *E. coli* O157: H7 despite high sensitivity of CHROMagar™STEC for this serotype [3].

### Clinical relevance of STEC carriage

On review of the clinical records of the five patients that carried STEC, none presented with typical Hemolytic Uremic Syndrome (HUS). However, the assessment of clinical relevance of STEC carriage was not possible due to the protracted time lag between the onset of sickness in the community and reporting to primary health care and eventual referral to tertiary health care. Earlier research has shown that only a small percentage of acute diarrhea cases in South Africa report to the health centres [28].

#### EAggEC, EPEC, and DAEC

At the GSH, stool from diarrhoea patients is not routinely screened for diarrhoeic *E. coli* other than non-sorbitol fermenting O157. Of all the 33 diarrhoeic *E. coli* strains isolated on CHROMagar™STEC, 45% (15/33) were EAggEC, 18% (6/33) were atypical EPEC, 15% (5/33) were typical EPEC, and 3% (1/33) were DAEC. No enteroinvasive *E. coli* (*E. coli*) were detected. This finding is in agreement with an earlier study which showed that EAggEC were most likely to be cultured from stool since they cause a more persistent form of diarrhea [14].

#### Serotypes

Of the 16 diarrhoeic *E. coli* serotypes that were isolated using CHROMagar™STEC in this study, only serotypes O111, O104 and O26 were previously reported to be detectable on this medium by studies conducted in Europe [3, 8]. In this study, we report the detection of the other tellurite resistant serotypes including O16, O175, O182, O186, O25, O3, O33, O175, O8, O9, O55, and O101. The dominant tellurite resistant diarrhoeic *E. coli* serotypes we identified in this study were O104 (15%), and O55 (18%). The cluster of six serotype O55 EPEC had clearly distinct antimicrobial resistance patterns and so was not an outbreak cluster. Since the cluster of five serotype O104 EAggEC strains were noted within a collection period of 38 days and had the same antimicrobial susceptibility pattern, they are possibly an outbreak cluster. However, we were unable to perform an epidemiological investigation to support this hypothesis.

#### Antibiograms

Even though diarrhoea is not normally treated with antibiotics (except if accompanied with invasive disease), *E. coli* is used for sentinel surveillance of antimicrobial resistance. The STEC detected in this study were only resistant to trimethoprim-sulphamethoxazole (SXT). This finding is similar to a 2011 study that was conducted in Kenya that reported a high level of resistance to SXT among STEC [29]. EAggEC, EPEC, and DAEC in this study showed resistance to SXT (100%, 28/28) and ampicillin (64%, 18/28). These findings are similar to reports from Kenya which showed a high prevalence of resistance to sulfamethoxazole among intestinal *E. coli* [30]. There are increasing reports of resistance to multiple antibiotics among EAggEC [31]. We identified multidrug resistance in one EAggEC (resistant to six antibiotics), one atypical EPEC strain (resistant to four antibiotics), and one typical EPEC strain (resistant to three antibiotics).

#### Sorbitol fermentation

Of the 33 diarrhoeic *E. coli* isolated on CHROMagar™STEC, 15 (45%) were non-sorbitol fermenting. A similar study conducted in Tanzania reported a 14% prevalence of non-sorbitol fermenting *E. coli* in 1049 human stool and non-human samples [32]. This is higher than the 2% (15/733) prevalence of non-sorbitol fermenting *E. coli* reported in this study. The high number of non-sorbitol fermenting EAggEC can be explained by the fact that serotype O104 is one of the prevalent non-sorbitol fermenting serotypes globally [3].

### Limitations of this study

CHROMagar™STEC only permits the growth of tellurite resistant STEC and not the tellurite susceptible strains. This study did not have a long sampling timeframe, and not all children that presented with diarrhoea may have had stool specimens taken. Only diarrhoeic *E. coli* that possessed virulence genes were characterised; therefore, we might have missed strains that lost the virulence genes.

## Conclusions

Given that clinical laboratories in Africa largely rely on screening for sorbitol negative STEC O157, the use of CHROMagar™STEC (with enrichment) would enable the detection of non-O157 STEC and other diarrhoeic *E. coli* pathotypes. However, more research is needed to figure out the extent of tellurite susceptible STEC which would be missed on use of this medium.

Additionally, further research is needed to characterise resistance to SXT and AMP in this region and to establish the clinical relevance of isolating STEC in the absence of a HUS or bloody diarrhea outbreak situation.

## Abbreviations

*aat*: Anti – aggregation protein transporter gene
AMK: Amikacin
ATCC: American type culture collection
AZT: Aztreonam
*bfp*: bundle forming pili gene
CAZ: Ceftazidime
CDC: Centre for disease control
CIN: Gentamicin
CIP: Ciprofloxacin
CLSI: Clinical Laboratory Standards Institute
COL: Colistin
CTX: Cefotaxime
*daaC*: fimbrial adhesion gene
DAEC: Diffusely adherent *Escherichia coli*
DOR: Doripenem
DOX: Doxycyclin
*eae*: intimin gene
EAggEC: Enteroaggregative *Escherichia coli*
EIEC: Enteroinvasive *Escherichia coli*
EPEC: Enteropathogenic *Escherichia coli*
ETEC: Enterotoxigenic *Escherichia coli*
ETP: Ertapenem
F: Female
FEP: Cefepime
GSH: Groote schuur hospital
HUS: Haemolytic uremic syndrome
IMP: Imipenem
*ipa*: invasive plasmid antigen
LEV: Levofloxacin
LT: Heat labile enterotoxin gene
M: Male
MEM: Meropenem
MIC: Minimum inhibitory concentration
MIN: Minicycline
NHLS: National health laboratory services
PCR: Polymerase chain reaction
POL: Polymixin
ST: Heat stable enterotoxin gene
STEC: Shiga toxin producing *Escherichia coli*
Stx: Shiga toxin gene
SXT: Trimethoprim - sulfamethoxazole
Ter: Tellurium resistance gene
TGC: Tigecycline
TIM: Ticarcillin – clavulanic acid
TOB: Tobramycin
TSB: Tryptic soy broth
TZP: Tazobactam - piperacillin
